# Definitions and Characteristics of Patient Digital Twins Being Developed for Clinical Use: Scoping Review

**DOI:** 10.2196/58504

**Published:** 2024-11-13

**Authors:** David Drummond, Apolline Gonsard

**Affiliations:** 1 Health Data- and Model-Driven Knowledge Acquisition Team National Institute for Research in Digital Science and Technology Paris France; 2 Faculté de Médecine Université Paris Cité Paris France; 3 Department of Pediatric Pulmonology and Allergology University Hospital Necker-Enfants Malades Assistance Publique – Hôpitaux de Paris Paris France; 4 Inserm UMR 1138, Centre de Recherche des Cordeliers Paris France

**Keywords:** patient simulation, cyber-physical systems, telemonitoring, personalized medicine, precision medicine, digital twin

## Abstract

**Background:**

The concept of digital twins, widely adopted in industry, is entering health care. However, there is a lack of consensus on what constitutes the digital twin of a patient.

**Objective:**

The objective of this scoping review was to analyze definitions and characteristics of patient digital twins being developed for clinical use, as reported in the scientific literature.

**Methods:**

We searched PubMed, Scopus, Embase, IEEE, and Google Scholar for studies claiming digital twin development or evaluation until August 2023. Data on definitions, characteristics, and development phase were extracted. Unsupervised classification of claimed digital twins was performed.

**Results:**

We identified 86 papers representing 80 unique claimed digital twins, with 98% (78/80) in preclinical phases. Among the 55 papers defining “digital twin,” 76% (42/55) described a digital replica, 42% (23/55) mentioned real-time updates, 24% (13/55) emphasized patient specificity, and 15% (8/55) included 2-way communication. Among claimed digital twins, 60% (48/80) represented specific organs (primarily heart: 15/48, 31%; bones or joints: 10/48, 21%; lung: 6/48, 12%; and arteries: 5/48, 10%); 14% (11/80) embodied biological systems such as the immune system; and 26% (21/80) corresponded to other products (prediction models, etc). The patient data used to develop and run the claimed digital twins encompassed medical imaging examinations (35/80, 44% of publications), clinical notes (15/80, 19% of publications), laboratory test results (13/80, 16% of publications), wearable device data (12/80, 15% of publications), and other modalities (32/80, 40% of publications). Regarding data flow between patients and their virtual counterparts, 16% (13/80) claimed that digital twins involved no flow from patient to digital twin, 73% (58/80) used 1-way flow from patient to digital twin, and 11% (9/80) enabled 2-way data flow between patient and digital twin. Based on these characteristics, unsupervised classification revealed 3 clusters: simulation patient digital twins in 54% (43/80) of publications, monitoring patient digital twins in 28% (22/80) of publications, and research-oriented models unlinked to specific patients in 19% (15/80) of publications. Simulation patient digital twins used computational modeling for personalized predictions and therapy evaluations, mostly for one-time assessments, and monitoring digital twins harnessed aggregated patient data for continuous risk or outcome forecasting and care optimization.

**Conclusions:**

We propose defining a patient digital twin as “a viewable digital replica of a patient, organ, or biological system that contains multidimensional, patient-specific information and informs decisions” and to distinguish simulation and monitoring digital twins. These proposed definitions and subtypes offer a framework to guide research into realizing the potential of these personalized, integrative technologies to advance clinical care.

## Introduction

Each industrial revolution has transformed the practice of medicine. The first 2 led to the development of new techniques for the industrial collection of new data (biological, imaging, etc) on the human body. The third—or digital—revolution transformed this analogue data into digital data, accelerating the exchange of information, and allowing the emergence of computer models to propose a diagnosis, establish a prognosis, and recommend a treatment [1-3].

For some contemporaries, we are currently in the midst of the fourth industrial revolution, which is the merging of the physical and digital worlds, based on 3 pillars: the internet of things, increasing connectivity, and machine learning–based decisions [4,5].

Digital twins are emblematic of this new industrial revolution. Grieves [6] introduced the concept in 2002 as a system consisting of a physical product, its virtual counterpart, and 2-way data exchange between the 2 entities. However, the term “digital twin” was first used and defined in 2010 by NASA (National Aeronautics and Space Administration) engineers as “an integrated multi-physics, multi-scale, probabilistic simulation of a vehicle or system that uses the best available physical models, sensor updates, fleet history, etc., to mirror the life of its flying twin” [7]. Following the popularization of the concept of the fourth industrial revolution in 2015, digital twins attracted interest from all industries [5]. With the objective of reducing production times through monitoring, coordination, and control of production systems, the manufacturing industry became the most active sector in terms of research and implementation of digital twins [8]. Digital twins also extended to construction, energy, transport, smart cities, agriculture, education, and health [8].

In the health sector, the concept of digital twins attracted interest from industry, scientists, clinicians, and patients [9-11]. Besides digital twins of hospitals, there is a strong rationale for the development of digital twins of patients, as these systems could offer personalized medicine through information gathered by the internet of things, real-time adaptation of treatments through efficient connectivity, and even automation of certain aspects of medical management through predictions based on machine learning [12]. An increasing number of scientific publications claim to be developing or to have developed “digital twins” of organs, physiological systems, or patients. However, when the term “digital twin” is used in this context, it remains unclear whether it corresponds to the definitions used in industry, or whether unique concepts and characteristics emerge for patient digital twins.

The 3 previous systematic literature reviews related to digital twins in health did not focus on patient digital twins [12-14]. To contribute to the understanding of patient digital twins, we conducted a scoping review to systematically map research in this area. The following research question was formulated: “What are the definitions and characteristics of digital twins provided by research teams claiming to develop patient digital twins for clinical applications, as reported in the scientific literature?”

## Methods

### Overview

We conducted this scoping review in accordance with the Joanna Briggs Institute guidelines for scoping reviews [15-17] and reported it following the PRISMA-ScR (Preferred Reporting Items for Systematic Reviews and Meta-Analyses extension for Scoping Reviews) checklist (Multimedia Appendix 1) [18]. The authors received training on the methodology of scoping reviews using the Joanna Briggs Institute Reviewers’ Manual 2020 [19]. We registered the protocol for this review at the Open Science Framework [20].

### Search Strategy

We created search strategies with a medical librarian to identify published papers on patient digital twins. Our initial search was conducted in MEDLINE via PubMed, using the terms “digital twin” or “digital twins,” combined with a broad range of medical-related terms pertaining to patients and precision medicine. We then adapted this search strategy for each included database and information source, namely, PubMed, Scopus, Embase, IEEE, Web of Science, and Google Scholar. When searching Google Scholar, we used incognito mode to minimize effects of past search histories and screened the first 500 results. Full search strategy is reported in Multimedia Appendix 2.

### Selection Process

We collated the identified citations from the comprehensive search and uploaded them into Rayyan, a web-based review tool [21]. Two independent reviewers (DD and AG) removed duplicates and screened titles and abstracts for inclusion. All screening was performed in a masked, duplicate fashion. Any disagreements between reviewers were resolved through discussion. Reasons for exclusion at the stage of full-text screening were recorded.

### Inclusion and Exclusion Criteria

We included peer-reviewed research papers in which the authors claimed to be developing or to have developed or tested a digital twin in health care, subsequently referred to as a “claimed digital twin” (CDT). Only literature published in English, French, or German was included up to August 31, 2023. Finally, to match the context of this review, we restricted included research to papers focused on developing or testing digital twins specifically representing patients, or components of patients.

Exclusion criteria were (1) animal studies, abstracts only, conference papers, reviews, editorials or correspondence, and non–peer-reviewed papers; (2) studies in languages other than English, French, or German; (3) studies unrelated to health; (4) digital twins not representing patients or part of a patient; and (5) studies limited to proposing a framework for a digital twin with no case study.

### Data Charting

Two reviewers (DD and AG) jointly developed a data charting form using Microsoft Excel to determine which variables to extract. For each included study, we extracted the following characteristics: study title, publication year, first author name, country of the corresponding author, and journal. We compiled the definitions of digital twins provided by the study authors and summarized their various dimensions (eg, digital replica, high-fidelity representation, and 2-way data exchange). Drawing from NASA’s digital twin definition, we assessed whether the developed digital twins were multiscale, integrated multiphysics modeling, and multiple data sources. We incorporated additional descriptors specific to digital twins for patient care, including medical discipline, organs or systems represented, categorization of whether the models constituted anatomical representations (eg, 3D) and models of physiological systems, and the study’s objective (simulation, prediction, monitoring, visualization, and generation of synthetic patients). Technical digital twin characteristics extracted included types of patient data used, approach used (mechanistic, data-driven, and hybrid), whether the digital twin included analytics and advanced visualization, constituted a model for simulations versus a simulation itself, capacity and frequency of updates, inclusion of none, 1-way or 2-way data exchange with the patient, and for 2-way exchange, the nature of feedback (recommendation or other type). Finally, we determined the clinical research phase of each digital twin by deriving the clinical research phases developed for artificial intelligence in medicine (phase 0: discovery and invention; phase 1: technical performance and safety; phase 2: efficacy and side effects; phase 3: therapeutic efficacy; and phase 4: safety and effectiveness) [22]. Additional information on each variable is given in Multimedia Appendix 2.

To ensure consistency, 2 reviewers (AG and DD) independently extracted data from the first 10 papers prior to full data extraction. Any disagreements were resolved through discussion. Following this pilot extraction and adaptation of the data charting form, we extracted data from the remaining studies in a masked, duplicate manner. Studies not meeting inclusion criteria were excluded. Any disagreements between reviewers were resolved through discussion.

### Unsupervised Classification of CDTs

To perform unsupervised classification of CDT, we analyzed dissimilarities by using the Gower distance metric on a subpart of our data set (the variables included are marked with an asterisk in Multimedia Appendix 2) [23]. The optimal cluster count was identified using the Partitioning Around Medoids method, guided by silhouette width analysis across 2-10 clusters [24]. We then used t-Distributed Stochastic Neighbor Embedding for dimensionality reduction to facilitate visualization in a 2D space. These analyses were performed in R (version 4.2.3) with the packages cluster, Rtsne, and ggplot2. We finally compared the characteristics of the CDTs of each cluster using the package gtsummary.

## Results

### Characteristics of Included Studies

**Figure 1:**
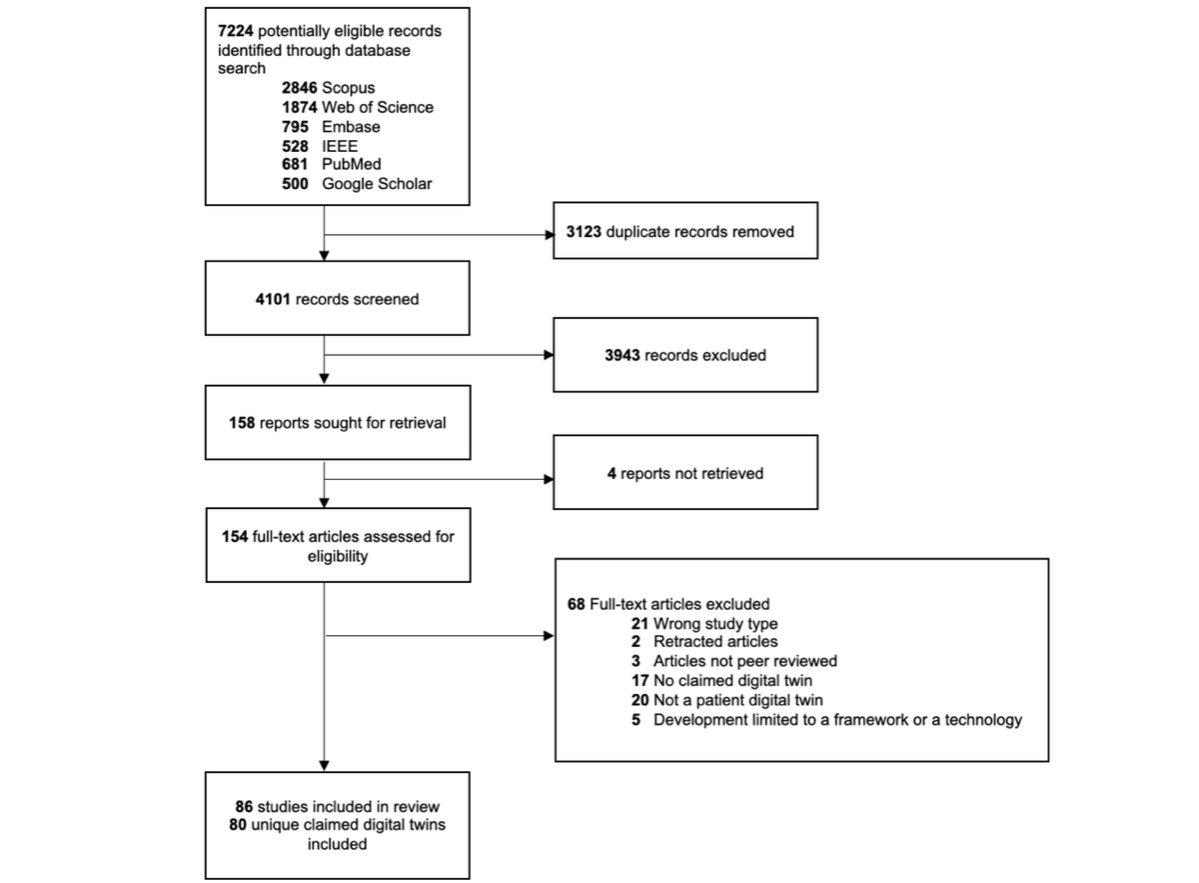
PRISMA (Preferred Reporting Items for Systematic Reviews and Meta-Analyses) flowchart.

A total of 7224 citations were identified from the search strategies (Figure 1).

After removing duplicates and screening titles and abstracts, 154 papers were examined in full text, and 68 papers were excluded (see Multimedia Appendix 2 for a full list of excluded full-text reviews with exclusion reasons). Finally, 86 papers [25-110] representing 80 unique CDTs were included in this scoping review. The full results, with individual data for each study, can be viewed in tabular form [20].

The first publications of CDTs appeared in 2019. Since then, publication volume increased yearly from 3 papers in 2019 rising to 23 papers in 2022 and 32 papers in the partial 2023 year (Multimedia Appendix 2). Although incomplete, 2023 totals through August 31 still reflect rising publication volume.

The included publications originated from 22 countries (Multimedia Appendix 2). The United States was the most common country of origin, with 25% (22/80 papers) of included publications. At the continental level, Europe was leading with 49% (43/80) publications, followed by North America with 29% (25/80) and Asia with 18% (16/80). No papers from South America or Africa were found.

### Definitions of Digital Twin Provided by Authors

**Figure 2:**
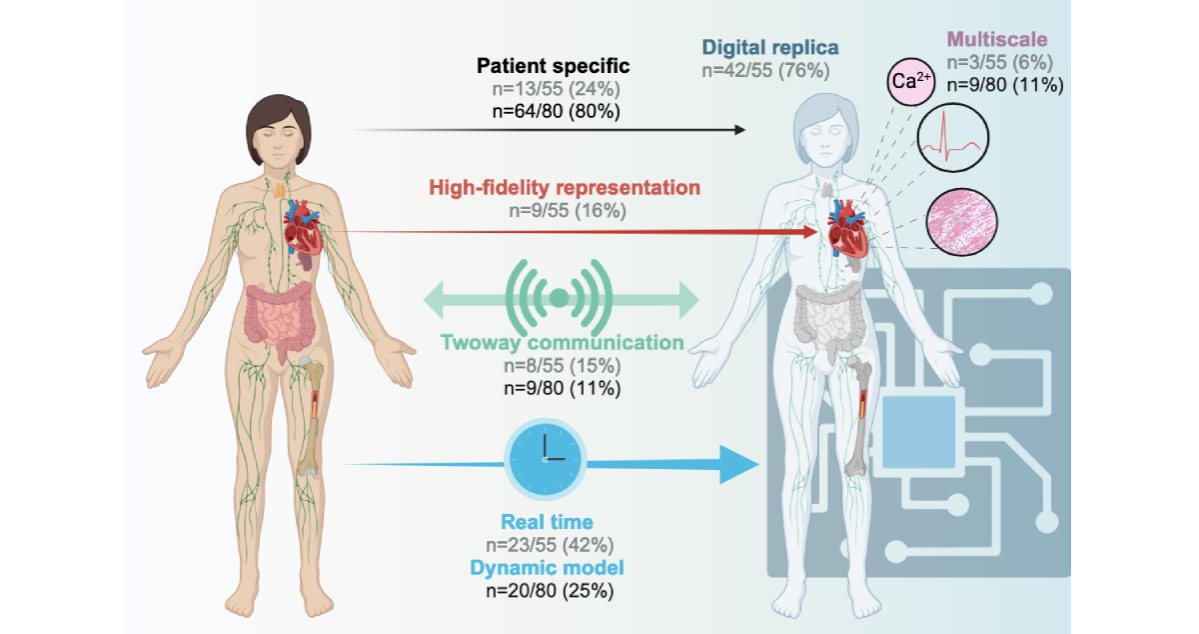
Dimensions of digital twins included in definitions provided by authors (gray) and included in the claimed digital twins (black). Created with BioRender [111] which is published under Creative Commons Attribution 4.0 International License [112].

Definitions of “digital twin” were provided in 63% (55/80 papers) of publications (Multimedia Appendix 2). Among papers with definitions, a digital twin was defined as a digital replica of a real object in 76% (42/55) of publications, with real-time update in 42% (23/55), a patient-specific approach in 24% (13/55), and a 2-way communication between the real and the digital object in 15% (8/55; Figure 2).

### Medical Domains

**Figure 3:**
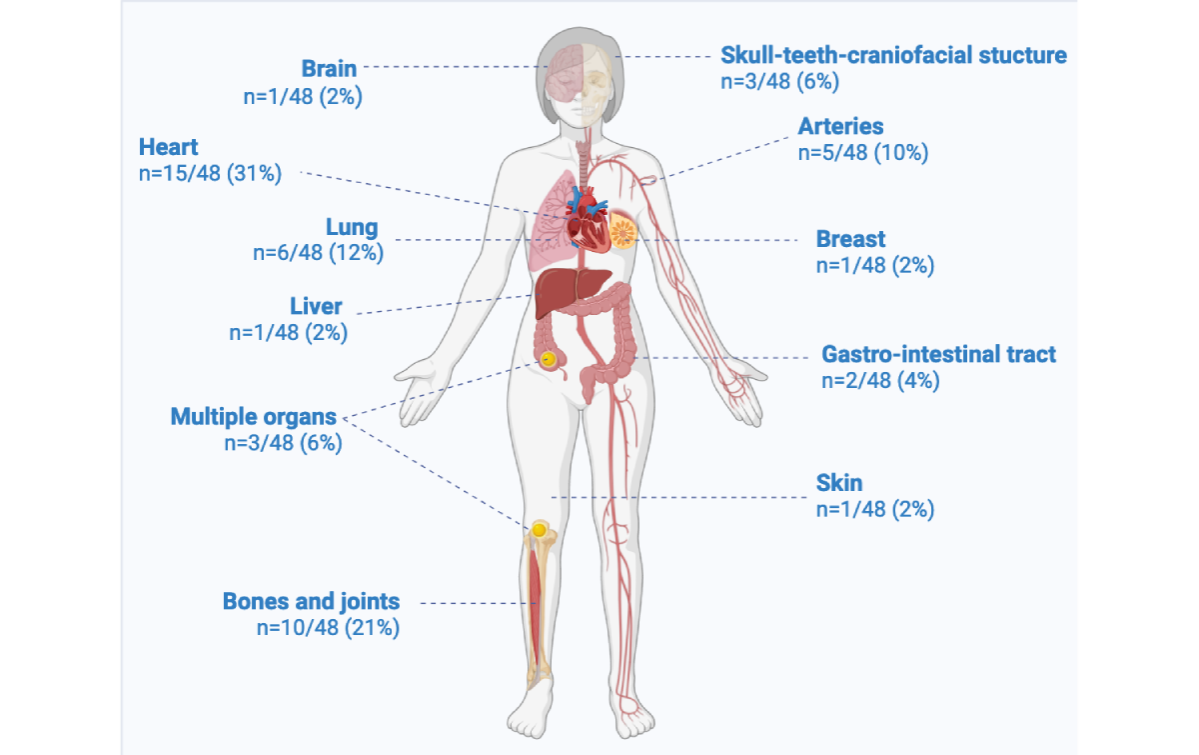
Distribution of organs or systems modeled in the claimed digital twins identified. N=48 claimed digital twin representing specific organs. Created with BioRender [111] which is published under Creative Commons Attribution 4.0 International License [112].

Of the 80 CDTs identified, 60% (48/80) represented specific organs or anatomical regions, while 14% (11/80) embodied biological systems (eg, immune system). The remaining 26% (21/80) of publications described other types of CDTs (eg, systems for emotion recognition, fetal heart monitoring, etc). Among the 48 publications with CDT representing specific organs, the most widely modeled organs were the heart (15/48 CDTs, 31%), the bones and joints (10/48, 21%), the lung (6/48, 12%), and the arteries (5/48, 10%; Figure 3 and Multimedia Appendix 2).

Approximately 6% (3/48) of CDTs involved multiple organs. Among the 11 CDTs representing biological systems, the endocrine (4/11, 36%) and the immune system (3/11, 27%) were the most widely involved (Multimedia Appendix 2). The most highly represented medical disciplines were cardiology (16/80 CDTs, 20%), oncology (10/80, 13%), and orthopedics (9/80, 11%; Multimedia Appendix 2).

### Characteristics of the Claimed “Digital Twins”

The patient data used to develop and run the 80 unique CDTs encompassed data from clinical notes (15/80 CDTs, 19% of publications), laboratory test results (13/80 CDTs, 16%), medical imaging examinations (35/80 CDTs, 44%), wearable device data (12/80 CDTs, 15%), and other data modalities such as electrocardiography, optical tracking, diet data, and intraoperative hemodynamic measurements (in 32/80 CDTs, 40%; Multimedia Appendix 2). Multimodal data incorporation, synthesizing various data types and sources, was present in 49% (39/80) of CDTs. Nearly all CDTs (78/80, 98%) involved data analytics and 36% (29/80) involved some form of advanced visualization. Moreover, 10% (8/80) of CDTs involved multiphysics and 11% (9/80) were multiscale. In terms of implementation, 50% (40/80) of CDTs followed a mechanistic approach, 28% (22/80) followed a data-driven approach, 21% (17/80) combined both approaches, and 1% (1/80) none of these approaches. Most of the CDTs developed (56/80, 70%) corresponded to models for simulation, that is, entities (organ and biological system) that can be used to simulate different states.

In terms of medical approach, 12% (10/80) of CDTs were categorized as anatomical, 28% (22/80) as physiological, and 35% (28/80) combined anatomical and physiological features. The objectives of CDTs were prediction (61/80, 76% CDTs), simulation (52/80, 65% CDTs), monitoring (11/80, 14% CDTs), visualization (10/80, 12% CDTs) and generation of synthetic patient data (8/80, 10% CDTs).

Among the 80% (64/80) of total patient-specific CDTs, 69% (44/80) were static models, whereas 31% (20/80) were designed as dynamic models with regular data inputs or outputs on a daily, hourly, or real-time basis (Multimedia Appendix 2).

Data flow topology characterizes the exchange of information between patient and digital twin. Moreover, 16% (13/80) of CDTs involved no flow from patient to their virtual counterpart, 73% (58/80) used 1-way flow from patient to their virtual counterpart, and 11% (9/80) enabled 2-way data flow between patient and their virtual counterpart (Figure 2). Within the nine 2-way digital twins, automated feedback occurred via recommendations to the patient or the physician in 6 cases and via direct feedback during surgical navigation in 3 cases.

Finally, regarding the clinical research phase of the CDTs, 98% (78/80) were categorized as phase 0 or 1 (preclinical phases) and only 2% (2/80) involved clinical assessments and thus reached phase 2. No CDT corresponded to phase 3 or 4.

### Unsupervised Classification of CDTs

**Figure 4:**
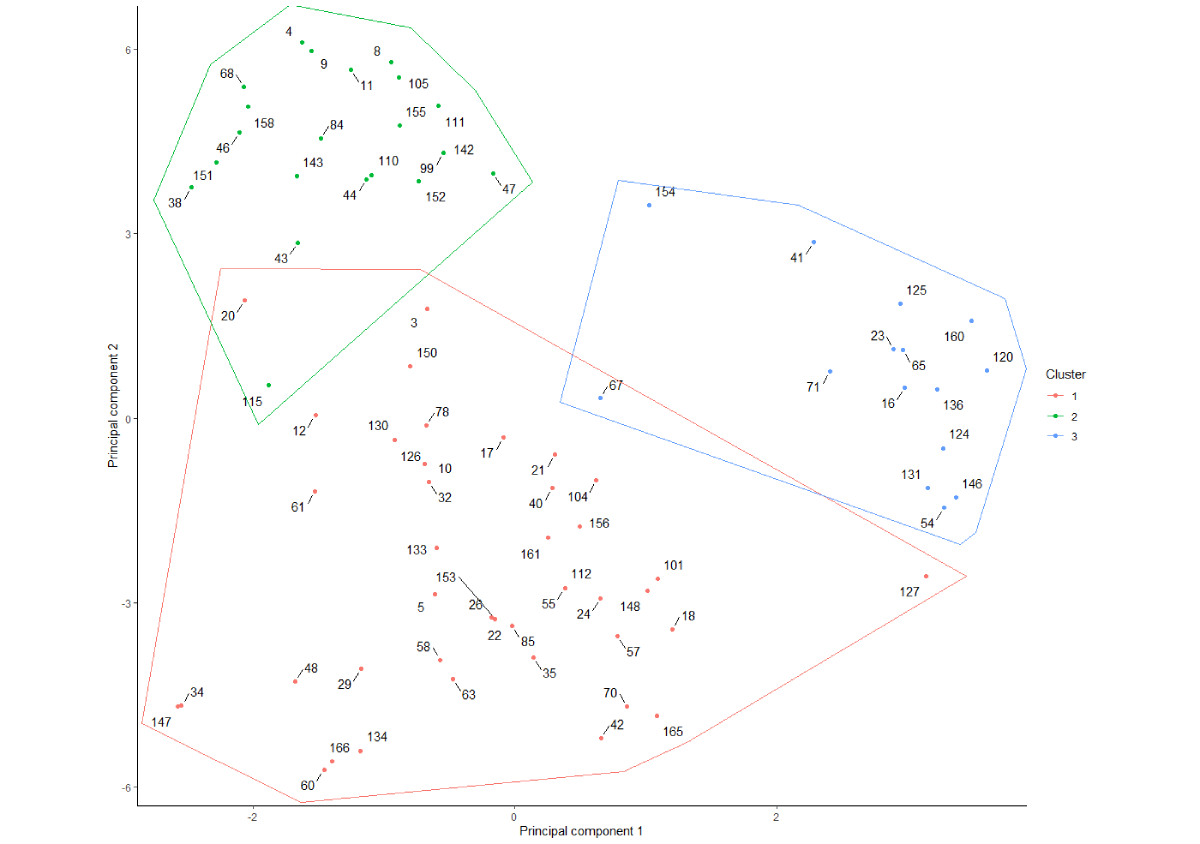
Clusters of claimed digital twins identified. Each point and its associated number correspond to 1 study and its study identification number. Cluster 1: simulation digital twins; cluster 2: monitoring digital twins; cluster 3: research-oriented models.

Three clusters were identified using the Partitioning Around Medoids method (Figure 4 and Multimedia Appendix 2). The highest average silhouette width was observed for a 3-cluster solution (average silhouette width=0.39), indicating that 3 clusters provided the best separation and cohesion for our data.

Cluster 1 included 43 unique CDTs, which corresponded to patient-specific organs or systems models for simulations and predictions, which we will refer to as “simulation digital twins.” Indeed, 98% (42/43) were models to perform simulations, of an organ or system, and these were anatomical and physiological models in 100% (43/43) cases relying on a mechanistic approach in 95% (41/43) cases. The output of these CDTs was patient-specific in 98% (42/43) cases, and their use was planned for onetime use in 84% (36/43) cases rather than for dynamic use.

Cluster 2 included 22 CDTs and mainly corresponded to prediction models and monitoring systems, which we will refer to as “monitoring digital twins.” In this category, the CDT most often did not involve an anatomical or physiological model of the patient (18/22, 82% CDTs). The approach was mainly data-driven (20/22, 91% CDTs). These CDTs were often dynamic (13/22, 59% CDTs) and provided feedback in 23% (5/22) of cases.

Cluster 3 included 15 CDTs and usually corresponded to models not linked to patients but designed for research, which we will refer to as “research-oriented models.” Indeed, in this group, only 7% (1/15) of CDTs were patient-specific [67]. Most CDTs were models for simulation (12/15, 80%) and involved a mechanistic approach (9/15, 60% CDTs). Examples of such CDTs are models of the immune response to vaccines [66] but also CDTs that corresponded to the generation of synthetic patients for in-silico trials [107] (5/15, 33% CDTs).

## Discussion

### Principal Findings

This scoping review highlights the growing interest in the concept of patient digital twins while at the same time revealing the lack of a uniform vision of this same concept among research teams.

The publication of scientific papers on digital twins across all sectors has experienced exponential growth, starting from 1 paper in 2014 to more than 1000 papers per year in 2022, predominantly in the manufacturing industry [113,114]. Studies focusing on the development and evaluation of patient digital twins emerged later, from 2019 onward, but have seen the same growth, particularly in Europe and North America.

### Lack of Consensus in Definitions of Digital Twins and Patient Digital Twins

Despite growing interest, there is no consensus definition of the digital twin concept. Depending on the research teams and fields of application, the definitions and characteristics of digital twins differ [114], and this is also reflected in our study, with a diversity of definitions and types of CDTs.

If one were to strictly import the digital twin definition used by Grieve [6] and NASA [7] from the manufacturing industry and aerospace sector to patients, a digital twin of a patient would be an integrated multiscale, probabilistic simulation of a patient that uses the best available models, sensor updates, medical history, and so forth, to mirror the health status of the patient in real time and act on them. But this definition is not currently achievable since no patient digital twin is able to capture the complexity of the human body in real time. Indeed, creating a patient’s digital twin is different from creating a digital twin of an object such as an airplane. An airplane is designed and developed entirely by humans, who have mastery over the composition and physical properties of each part of an airplane, the assembly of all these parts, and the interaction of the whole. In contrast, we still have an imperfect understanding of the functioning of the human body and the interaction of its different organs and systems. It is therefore simpler to model an airplane than the human body with a high degree of fidelity and in a multiscale way. It is also easier to integrate additional sensors and actuators on an airplane’s controls than to have patients continuously wear or have implanted sensors or actuators to obtain the 2-way, real-time data exchange between the plane or patients and their digital twin. Moreover, while the digital twin of the airplane relies essentially on the physical properties of the aircraft, the digital twin of a patient will have to go beyond anatomical and physiological models of the patient’s various organs and systems to include models of the patient’s cognitive and emotional functioning [115]. Finally, concerning the practical use of the term “patient digital twin,” they must be able to echo patients’ own perceptions of what a digital twin is. These terms are not neologisms, but each refers to concepts already used in everyday language by patients. Because twin refers to a multiple birth, it is likely that the patients picture a digital twin as a realistic avatar of themselves in the same health state. This implies that in the mind of a clinician or patient not well versed in computer science, the first representation of a patient digital twin will be that of a 3D representation of the patient’s body and its functioning.

### A Practical Definition for Patient Digital Twins

For all these reasons, the concept of a digital twin, which is appropriate in manufacturing, could be considered inappropriate in medicine. However, on the basis of the various papers in this review, we believe that this concept actually corresponds to current advances brought by the multiplication of data sources, data analysis, and the visualization of patients’ state of health. It could also be a useful educational tool for the communication with patients when discussing models and predictions made from their data. Since it is currently not feasible to bring together all the characteristics of the digital twin in manufacturing, we propose the following definition of a patient digital twin: “A patient digital twin is a viewable digital replica of a patient, organ, or biological system that contains multidimensional, patient-specific information and informs decisions.” This definition is based on the characteristics of the digital twins identified in this review (digital replica, multidimensional, and patient-specific) and aligns with the broader definition of the National Academies of Sciences, Engineering, and Medicine, which defines a digital twin as “a set of virtual information constructs that mimics the structure, context, and behaviour of a natural, engineered, or social system (or system-of-systems), is dynamically updated with data from its physical twin, has a predictive capability, and informs decisions that realize value” [116].

The advantage of our definition is that it can exclude what is not a patient digital twin: generic models of cells, tissues, organs, or biological systems not linked to a patient but used to study disease progression or drug development [33,58,84], which corresponded to our cluster 3 (research-oriented models); pure cyber-physical systems, that is, systems such as implantable cardioverter-defibrillators, or artificial pancreas, which do not use a representation of the patient and therefore not a “viewable” digital replica of the patient; digital patient data created from patient databases for in-silico trials [51,103,107]; often using generative adversarial networks, which we propose to call “synthetic patients” instead; data sets from another patient, similar to those of the index patient [81]; machine learning–based classifiers, trained on a population to predict a diagnosis [73]; and patient models built from a single data source, such as demographic characteristics or imaging [35,51,53].

### Simulation and Monitoring Patient Digital Twins

**Figure 5:**
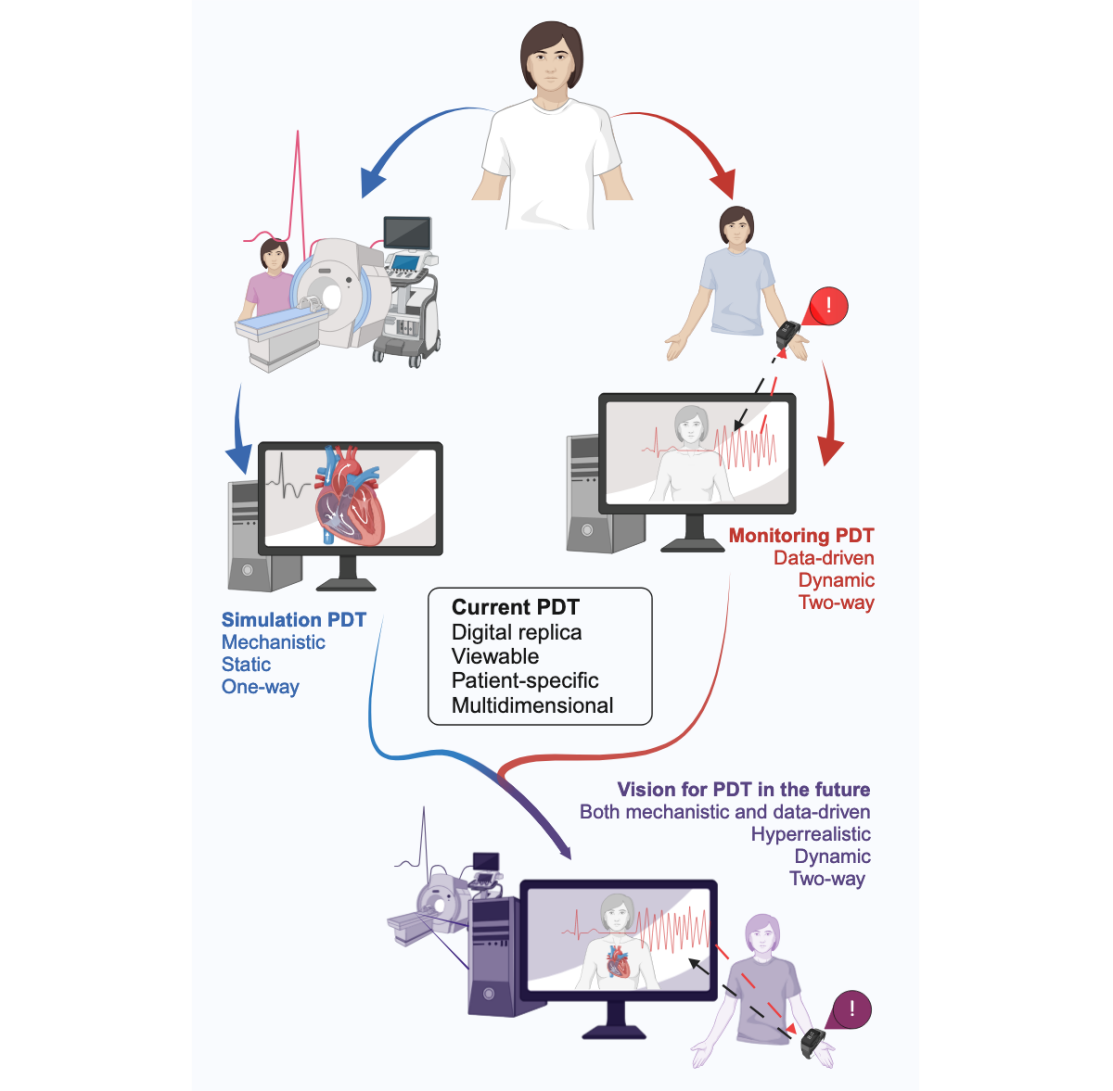
Proposed definitions and characteristics of patient digital twins. PDT: patient digital twin. Created with BioRender [111] which is published under Creative Commons Attribution 4.0 International License [112].

This definition of patient digital twins also encompasses the 2 major trends revealed by this study. On one hand, digital twins offering a high degree of fidelity, combining advanced anatomical and physiological models, based on mechanistic approaches or combining mechanistic and data-driven approaches [47,70,109]. However, their limitation is their common reliance on hospital-acquired data such as imaging data (computed tomography and magnetic resonance imaging) or intraoperative hemodynamic data. They are therefore generally restricted to onetime purposes and not in a dynamic form. On the other hand, digital twins corresponding to real-time representations of patients through home-based data collection via wearable, using machine learning techniques for analysis and alert detection [29,31]. This type of digital twin is dynamic, can be 2-way by giving recommendations to patients or by modifying a biological parameter of the patient via medical devices, but often offers little or no information on the patient’s anatomy or physiology. For it to be differentiable from a cyber-physical system or a telemonitoring system, it must integrate different data sources, data analysis systems, and a visualization of the patient organ, system, or body. We thus propose 2 major categories of patient digital twins based on our clustering analysis (Figure 5).

Simulation patient digital twins (derived from cluster 1): personalized, static, viewable digital replicas of patients’ anatomy and physiology based on computational modeling to run simulations predicting outcomes in hypothetical scenarios or evaluating therapeutic approaches. These digital twins are mostly used for onetime assessments rather than continuous monitoring.

Monitoring patient digital twins (derived from cluster 2): personalized, dynamic, viewable digital replicas of patients leveraging aggregated health data and analytics to enable continuous predictions of risks and outcomes over time and provide feedback for optimizing care. These digital twins are mostly focused on continuous tracking rather than detailed mechanistic simulation.

An emblematic example of a simulation patient digital twin is a personalized cardiac model that integrates a patient’s anatomical and physiological data to create a 3D virtual representation of his or her heart [61]. Computational simulations are then performed on this digital twin to predict outcomes or optimize treatments, such as evaluating different ablation strategies for atrial fibrillation. These digital twins enable onetime, patient-specific assessments through detailed mechanistic modeling.

An emblematic example of a monitoring patient digital twin would be a personalized representation of the bronchi of a child with asthma based on real-time data from connected objects such as smart inhalers, spirometers, and portable air quality monitors [117]. This may allow prediction of the risk of an asthma attack and enable children to visualize the targets of the asthma treatments they are taking, namely, bronchial inflammation and bronchoconstriction.

### Future Directions

As technical progress is made, it is likely that these 2 types of patient digital twins will converge, combining both mechanistic models and increasingly efficient continuous data collection and data-driven approaches [118]. However, currently, only 14 CDTs from this review would meet the definition of patient digital twins combining multidimensional, patient-specific data, data analytics, and advanced visualization, all being simulation patient digital twins [27,38,41,47,52,61,62,65,70,74,92, 102,109,110]. Digital twins for surgical navigation systems are the most advanced, with 3 teams presenting patient digital twins combining both a mechanistic approach and a dynamic real-time adaptation [62,92,110].

The main benefit expected from these patient digital twins is to offer increasingly personalized medicine, taking into account all the available data and patient-specific simulations and predictions. This would make it possible, for example, to choose the most appropriate drug treatment based on the patient’s medical history, allergies, comorbidities, and genetic profile [26]; the most appropriate stent for the configuration of the aortic dilatation [27]; and the least risky surgical approach thanks to preoperative simulations. But the benefits could also be for patient education and engagement: patients would interact with their digital replica to better understand their own body, health conditions, and influence of behaviors, and collaborate more with providers.

### Strengths and Limitations

The strength of this review is to be the first to have carried out a systematic analysis of the literature concerning patient digital twins and to have been able to identify the major trends. We also acknowledge several limitations. First, in the absence of a consensual definition of the digital twin concept, we chose to include all papers in which the authors claimed that they had developed a digital twin or part of a digital twin. This approach undoubtedly led us to exclude some papers that embody the concept of a digital twin but did not use that specific term, such as works from the Physiome Project [119] or the Virtual Physiological Human initiative [120]. Conversely, it may have resulted in the inclusion of some papers that, in fact, did not develop a digital twin but only a simple prediction model. To mitigate this limitation, we proposed our own definition and typology of patient digital twins, derived from characteristics found in the reviewed health care literature and aligned with well-established definitions from other fields, such as NASA’s definition. This approach ensures that our definition is grounded in current health care applications and fundamental digital twin principles, enhancing its robustness and adaptability.

Second, we realized that some characteristics of digital twins were not appropriate for consistent assessment, either because they were too industry-specific, such as the “multiphysic” characteristic, or because they did not allow for consistent categorization. For example, we often struggled to determine whether a presented digital twin was at phase 0 or phase 1 of development and chose to merge these 2 categories into a single “preclinical” category. This may have affected the granularity of our analysis. Future research could develop more precise and universally applicable criteria for classifying digital twin characteristics.

Third, we did not include papers written in languages other than English, French, or German. This language limitation may have led to underrepresentation of work from regions where other languages are predominant, potentially biasing our findings toward research conducted in these languages.

Fourth, we did not include conference papers. This exclusion might have omitted recent or preliminary findings that are often first presented at conferences, possibly affecting the comprehensiveness of our review.

Finally, our cluster analysis has limitations due to potential sensitivity to variable selection and the choice of clustering methods, which may influence the results. In addition, the relatively small sample size of 80 CDTs could affect the stability and generalizability of the clusters identified. Although we carefully selected variables and methods, these factors may still influence the findings. Therefore, our analysis provides valuable insights, but these should be interpreted with caution. Future research with larger sample sizes and exploration of different clustering techniques could enhance the reliability and generalizability of such analyses.

### Conclusions

We propose that a patient digital twin be defined as “a viewable digital replica of a patient, organ, or biological system that contains multidimensional, patient-specific information and informs decision.” We currently identify 2 categories, simulation patient digital twins and monitoring patient digital twins. In the future, we envisage a fusion of these 2 types of digital twins that will combine a high degree of fidelity based on anatomical and physiological models with real-time updating and feedback to the patient.

## Appendix

Multimedia Appendix 1 PRISMA-ScR (Preferred Reporting Items for Systematic reviews and Meta-Analyses extension for Scoping Reviews) checklist.

Multimedia Appendix 2 Additional methods, tables and figures.

## Abbreviations

CDT: claimed digital twin
NASA: National Aeronautics and Space Administration
PRISMA-ScR: Preferred Reporting Items for Systematic Reviews and Meta-Analyses extension for Scoping Reviews

## Data Availability

The data related to the studies included in this manuscript can be found at on Open Science Framework [20].
